# The Impact of Racial and Low Socioeconomic Status on the Implementation of Spinal Cord Stimulation for Chronic Pain in the United States

**DOI:** 10.1007/s11916-024-01315-6

**Published:** 2025-01-30

**Authors:** Gabriel Howard, Luis Guinand, Eric Xu, Alex Kervyn, Behnum Habibi

**Affiliations:** Department of Physical Medicine & Rehabilitation, Temple University Hospital, Philadelphia, PA USA

**Keywords:** Spinal Cord Stimulation, Socioeconomic Status, Race, Low Back Pain

## Abstract

**Objectives:**

This study aims to review the societal, economic, and racial factors that impact the usage of spinal cord stimulation for chronic pain. Our working hypothesis is that patients of ethnic minority groups or of lower socioeconomic status (SES) status may have lower implantation rates and usage of spinal cord stimulation (SCS).

**Materials and Methods:**

Our study sourced publications from PubMed, Embase, and Cochrane Library on December 21st, 2023 for SCS for the purposes of pain management. Articles were excluded from the review if the study was not USA based, did not involve SCS for the purpose of pain or did not allow for the subgroup analysis. There were 1028 reports that resulted after the initial search with 184 duplicates which were removed. Six reports met the inclusion and exclusionary criteria and were included in the review.

**Results:**

Several trends were able to be extrapolated from the pooled reviews. Orhurhu et al. found that Black and Hispanic minorities had a higher utilization rate of SCSs than their White and Asian counterparts in the inpatient setting. Jones and Missios et al. found that in the outpatient setting, White and privately insured patients were more likely to utilize SCS. Ovrom et al. observed an increased cost associated with Hispanic ethnicity and inpatient SCS utilization. Wondwossen et al. found that in the US military system White patients were more likely to receive SCS earlier in their care than Black patients. Labaran et al. concluded the Southern US completed more SCS implants, particularly in White patients with Medicare insurance.

**Conclusions:**

White patients are recipients of SCS earlier and more frequently than minority patients in the outpatient setting. There is mixed evidence regarding inpatient SCS and how household income relates to SCS usage. Insurance type and coverage may be more accurately predictive than simple household income for SCS utilization.

## Introduction

There is an abundance of studies that have investigated the ethnic and socioeconomic disparities that exist when accessing the healthcare system in the United States and the impact of these disparities on healthcare outcomes [1, 2]. Stepanikova et al. observed that socioeconomic status (SES) was an important social determinant of perceived privilege and discrimination in healthcare settings. Multiple demographic groups, particularly individuals who face cost-related barriers to healthcare were at increased risk of perceived discrimination. The impact of low socioeconomic status and ethnic disparities within the field of pain medicine has also been elucidated extensively in the literature [3, 4]. Janevic et al., using the nationally representative Health and Retirement Study (HRS) respondents consisting of adults older than age 50, found that rates of disabling chronic pain rose among individuals in the lowest wealth quartile [3]. It was also noted that for adults with any chronic pain, individuals in the lowest wealth quartiles and African Americans reported more pain-related disabilities across multiple domains such as family/home, leisure, social, work, and activities of daily living [3].

A growing area of focus within pain medicine is the impact of low socioeconomic status and racial disparities related to implantation and associated costs of spinal cord stimulators [5]. Despite this growing area of interest, the actual body of literature remains scarce. This is particularly true when investigating how ethnicity and low SES among patients in the US population may affect access to pain interventions as a therapeutic option in chronic pain syndromes. In recent decades, there has been a notable paradigm shift regarding the treatment of chronic pain. Given the side-effect profile and nationally alarming rates of misuse of chronic opioids, there has been an advent and need for more sustainable approaches to the treatment and management of chronic pain. One of the alternative approaches is the use of neuromodulation, a subtype of which is spinal cord stimulation (SCS). SCS serves as a viable non-pharmacologic treatment for patients with refractory chronic pain [6, 7]. Common indications for the implementation of SCS include chronic back pain, complex regional pain syndrome (CRPS), post-laminectomy syndrome (PLS), limb ischemia, angina, and peripheral neuropathy [6, 8].

The use of SCS as a therapeutic alternative is widely based on the Gate Control Theory of Pain developed by Ronald Melzack and Patrick Wall in 1965. This theory postulates that electrical stimulation of Αβ fibers in the dorsal column tract of the spinal cord has the potential to modulate nociceptive signals that are transmitted by smaller Aδ and C fibers to the brain that transmit signals through the spinothalamic tract to the brain [7, 9]. The spinothalamic tract serves to relay pain, touch and pressure signals to the brain after receiving sensory input from afferent nerve fibers. For patients interested in this treatment, there is a trial period to assess the response to SCS. If the patient has a significant and positive response, then a permanent stimulator is implanted. As a review, there are two main components to the SCS; the leads and implantable pulse generator [10]. While the leads of the SCS are always placed within the epidural space, the exact lead locations (e.g., cervical, thoracic, or lumbar placement) can be varied based on where the patient has pain. Meanwhile, the electrical pulse generator is placed under the skin of the abdomen, low back, or buttocks via a surgical incision [6].

For this study, the objective is to evaluate the relationship and trends of spinal cord stimulation implementation in populations with a low socioeconomic status in the United States by performing a systematic review of the literature currently available. Our working hypothesis is that patients who are of an ethnic minority group and/or of a lower SES status may show lower implantation rates and usage of SCS. We hope to further elucidate the possible disparities that are observed and contribute to the current literature regarding this topic.

## Materials and Methods

**Fig. 1:**
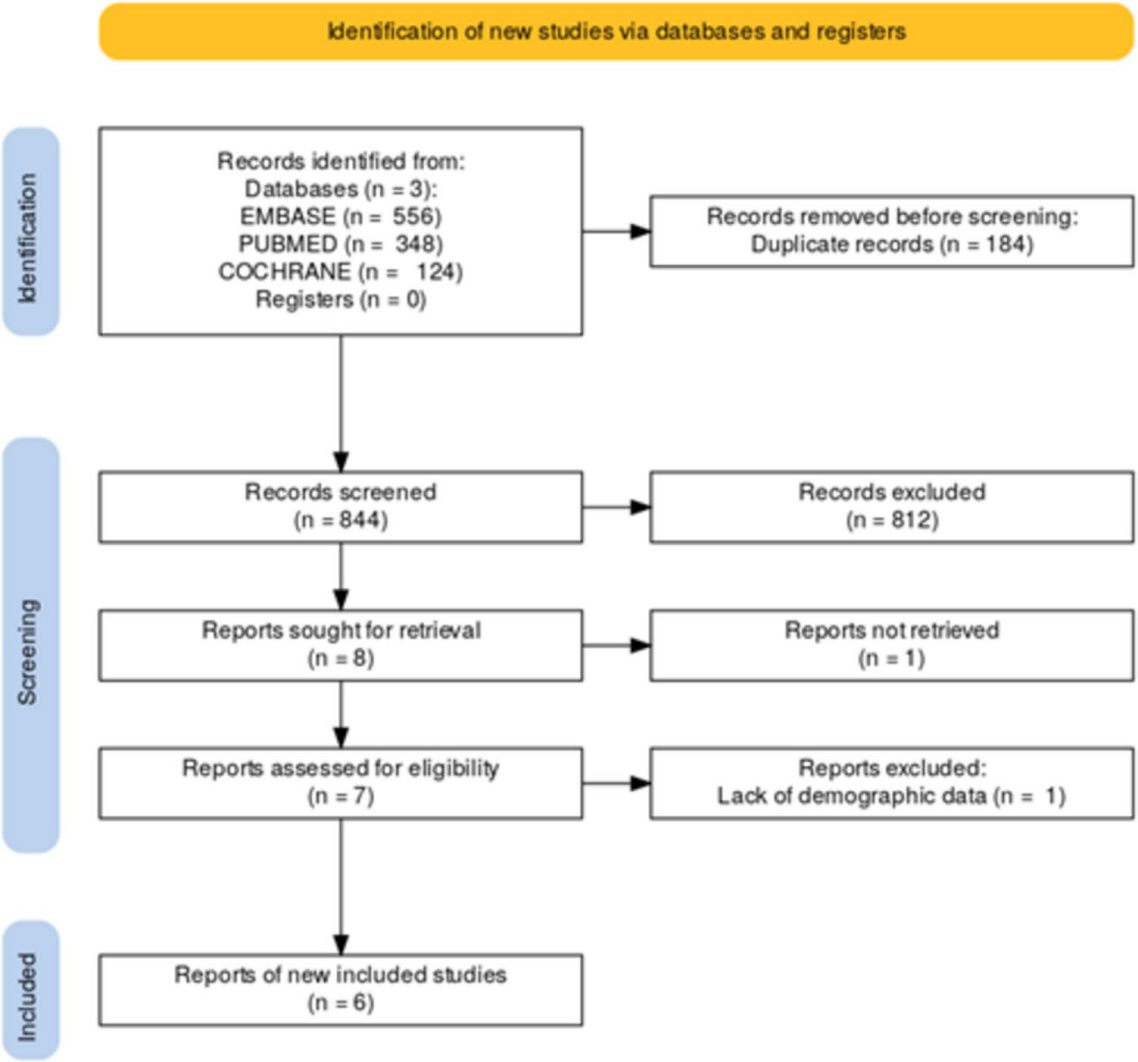
Diagrammatic representation of article screening and final selection for included studies described in the Materials and Methods section

Our study sourced publications from three of the largest medical databases including PubMed, Embase, and Cochrane Library. These databases are commonly used and trusted for the purposes of medical research and literature review. Our search was queried on December 21st, 2023 for all-time publication data based in the United States of America (USA). Search results must include the terms “Spinal cord Stimulation”, “Dorsal column stimulation”, and may include the terms “SES”, “Socioeconomic”, “Insurance”, “Medicaid”, “Medicare”, “Race”, “Ethnicity”, and “Gender”. Subsequent article review was predicated on strict inclusionary and exclusionary criteria which served as the basis for final literature review and discussion materials. For inclusion, articles must include SCS as the primary intervention and involve treatment of specified populations including those with low socioeconomic status, minority status, or otherwise underserved populations. The underserved populations receiving SCS must be specified with particular demographic data available for review. Furthermore, each group must be distinguishable from another in order to comment on differences of outcomes regarding treatment courses. Patients must be receiving SCS for the purposes of pain management, such as for CPRS, PLS or other pain syndromes. Articles were excluded from the review if the above criteria were not met, particularly if the study was not USA based, did not involve SCS for the purpose of pain or did not allow for subgroup analysis. Patients receiving SCS or any reason other than for pain management were excluded. Patients receiving electrical stimulation which was not distinctly SCS were also excluded, such as peripheral nerve stimulation, dorsal root ganglion stimulation, deep brain stimulation, transcutaneous stimulation and functional electrical stimulation. There were 1028 reports that resulted after the initial search with 184 duplicates which were removed. Two blinded reviewers performed title and abstract review screening the remaining 844 reports for eligibility with conflicts resolved by a third unblinded reviewer. Eight reports met the inclusion and exclusionary criteria and were sought for retrieval. Following full report retrieval, one article was removed due to lack of reported demographic data and the final article was removed due to inaccessibility of the full text. See Fig. 1 for diagrammatic breakdown of screening results.

## Results

Orhurhu et al. analyzed the National Inpatient Sample (NIS) from the years 2011 to 2015 which utilized hospital inpatient billing information for 40,858 patients admitted with the primary problems of “Failed Back Surgery Syndrome” and “Complex Regional Pain Syndrome Type 1” (CRPS1) [11]. The authors searched these admissions for SCS use and additionally for medical comorbidities, race, and income (by extension of zip code). After multivariate logistic analysis, the study found that Black and Hispanic minorities had a higher utilization rate of SCSs for Failed Back Surgery Syndrome and CRPS 1 than their White and Asian counterparts. There was no statistical difference in utilization of SCS among Asian/pacific islanders and Whites. The Black population studied was found to be more likely to be in the bottom quartile of annual income and have a higher burden of medical comorbidities. The authors also concluded that Medicaid recipients and self-pay patients were less likely to receive SCS than Medicare recipients. However, privately insured patients were more likely to utilize SCS than Medicare recipients. Household income was not independently associated with statistically significant utilization of SCS. Findings from this analysis seem to indicate that in the inpatient population, Black and Hispanic populations are utilizing SCS at a higher rate than their White and Asian counterparts. Similarly, privately insured and self-pay patients are utilizing SCS at a higher rate than their Medicare counterparts regardless of household income. The cause of these discrepancies was not definitively linked to any one isolated variable, however is likely due to many factors that may be difficult to determine. Many variables were unable to be included in this study due to lack of availability within the NIS including the severity of Failed Back Surgery Syndrome/CRPS I, literacy or preconceived preferences regarding SCS, whether or not SCS was offered at different rates between certain groups and other considerations which may affect SCS utilization. The study was also limited by presenting inpatient data only which is in contrast to the typical outpatient SCS encounter and implantation.

Jones et al. conducted a retrospective study using data from the Medicare Limited Data Sets (LDS) including 1,244,927 unique cases to determine racial and socioeconomic trends in SCS implantation [8]. The study searched Centers for Medicare and Medicaid Services (CMS) supplied data from LDS for the diagnoses of post-laminectomy pain syndrome (PLS) and chronic pain syndrome (CPS) with subsequent extraction of demographic data and SCS use. Data was extracted from outpatient encounters from the years 2016–2019. The authors found that compared to White patients, all minority ethnic groups were less likely to receive SCS procedures for either diagnosis. Those with dual eligibility of Medicare and Medicaid were also found to receive SCS less frequently than Medicare alone. Multivariable analysis was performed to adjust for all associated factors aside from race and the discrepancy remained with White patients being more likely to receive SCS when compared to Black, Asian, Native American and Hispanic patients. Further, it was found that patients with some comorbidities such as anxiety, intellectual impairment, anxiety were less likely to receive SCS while patients with others such as diabetes, depression, nicotine dependence, and obesity were more likely to receive SCS than those without them. A secondary multivariable analysis showed that there was no combinatory effect of being a minority race and being Medicaid eligible. A proposed etiology for this discrepancy is that minorities may receive lower quality care than their White patients, leading to not only additional comorbidities, but also more significant medical conditions. As a result, this higher comorbidity burden may prevent minority group patients from being candidates for SCS as frequently as their majority counterparts. However, even after controlling for comorbidities, the results of this study suggest that other factors including racial bias may be involved. A significant strength of this study is the robust nature of the Medicare database and that all patients were observed in the outpatient setting. Because most SCS is performed in the outpatient setting, this metric may be more useful to physicians in this domain. The study is limited to Medicare and Medicaid patients only; therefore no assumptions could be made about self-pay or privately insured patients. Additionally, there was no longitudinal data that may provide insight into outcomes associated with SCS nor any data that documented the rate at which SCS was offered to patients and subsequently declined.

Ovrom et al. completed a retrospective cohort study which examined length of stay (LOS) and cost variations between race, region and household income following SCS surgery [6]. The index of data used was from the National Inpatient Sample (NIS) from the years 2016–2018 with 3351 cases included in the analysis. The authors found that most SCS recipients were White females with a mean age of 56.4 years old. White patients were older at the time of SCS surgery than their minority counterparts. SCS was more likely to be implanted in the Southern United States (as defined by the authors) at urban teaching hospitals. SCS was implanted evenly among White patient economic demographics, however Black and Hispanic populations were more likely to receive SCS in the lowest economic quartile. Most patients receiving SCS were Medicare insured, which was consistent across all racial groups. The study did not assess the impact of insurance on relative likelihood to receive SCS between insurers. After adjusting for age, hospital region, gender, surgical approach, insurance, comorbidities and medical complexity, the authors found a strong association with Hispanic ethnicity and increased cost of inpatient SCS when compared to White and Black patients. Although more Hispanic patients were from the Western United States region than any other demographic, the higher costs associated with this reason were not solely accountable for the discrepancy. The authors proposed that Hispanic patients may have less access to preventative treatment modalities and may be more likely to experience language barriers as well as cultural barriers which may lead to increased costs of SCS.

Missios et al. performed a retrospective cohort study looking at demographic trends of patients who received either inpatient or outpatient SCS implantation in New York, Florida, California and North Carolina from 2005 to 2008 through the State Inpatient Database (SID) and State Ambulatory Surgery Databases (SASD) [5]. There were 4843 patients who underwent outpatient SCS implantation and an additional 4197 who underwent inpatient SCS implantation included in the trial. This study sourced data from the State Inpatient Databases and State Ambulatory Surgery Databases for patients that underwent inpatient and outpatient SCS placement, respectively. The primary variables assessed were sex, race, insurance type (Medicare/Medicaid, private), income (quartiles), and hospital type (quartiles of inpatient vs outpatient procedure load). Multivariable analysis was performed to assess the independent effect of each variable on the implementation of SCS. It was found that White, male, privately insured patients were more likely to receive SCS, particularly in high volume outpatient hospitals. Those with Medicare/Medicaid insurance and those with a higher degree of comorbidities were significantly less likely to receive SCS.

Wondwossen et al. conducted a retrospective cohort study of active-duty and retired military members with the diagnosis of persistent spinal pain syndrome (PSPS) following spinal surgery [12]. Patient information was obtained from the US Military Health System from January 2017 to January 2020 with 8807 total cases reviewed. Following multivariable analysis, the primary result was that White patients with a diagnosis of PSPS were more likely to receive SCS than Black patients. There was an inverse correlation noted between the number of spine procedures such as epidurals, radiofrequency ablations, acupuncture, and botulinum injections that were received and the likelihood of SCS receipt. Between White and Black patients who had received less than three spine procedures, White patients were significantly more likely to undergo SCS treatment than Black patients. However, White and Black patients who had undergone greater than three spine procedures had no significant differences in SCS utilization. The trial was limited to the American military population only and found no statistical difference between active and retired military members for likelihood to receive SCS.

Labaran et al. analyzed demographic trends in paddle lead SCS placement through PearlDiver Records Database which included 34,287 device implants [13]. Medicare (*n* = 31,352) and private-payer (*n* = 2,935) insurances were included in the database as well as demographic information such as region (Midwest, Northeast, South, West), sex, and race. Data was adjusted to reflect implants per capita which demonstrated that the majority of SCS therapy was utilized in women and the Medicare populations when compared to men and those who were privately insured. Those in the “South” region and of White race also received more SCS therapy, however no multivariable regression was performed to assess the significance of variables of sex, race or region alone. This demographic data is helpful in understanding population level utilization of paddle lead SCS and reports the unique variable of region specific data. The study also benefits from a large sample size with mixed insurer data including both Medicare and privately insured. The study is limited in its application because there was no assessment of individual variable significance and therefore impossible to determine if any one variable specifically was associated with higher SCS use. Further statistical analysis could be performed to better understand the data collected. Summary of all included studies are outlined in Table 1.

**Table 1:** Summary of included studies with database, setting, demographics and key findings listed

	Database and years sampled	Database Setting	Demographics/Factors assessed	Key finding
Orhurhu et al	National Inpatient Sample (NIS) 2011 to 2015	Inpatient	Race, Age, Household income, Comorbidities, Insurance Coverage (Medicare/Medicaid/Private/Self pay)	Black and Hispanic individuals had a higher utilization rate of SCSs than other demographics in the inpatient setting
Jones et al	Medicare Limited Data Sets (LDS) 2016 to 2019	Outpatient	Race, Age, Medicare/Medicaid eligibility, Comorbidities	Factors such as Dual Medicare/Medicaid eligibility, minority race, and comorbid anxiety were associated with less SCS implants in the outpatient setting
Ovrom et al	National Inpatient Sample (NIS) 2016 to 2018	Inpatient	Race, Age, Median Household income, Insurance Coverage (Medicare/Medicaid/Private/Self pay), Hospital Region	Hispanic patients had higher charges than other racial demographics for inpatient SCS surgery and no disparity in charge was found between White and Black patients
Missios et al	State Inpatient Database (SID) and State Ambulatory Surgery Databases (SASD) from New York, California, Florida, North Carolina 2005 to 2008	Inpatient and Outpatient	Inpatient vs outpatient status, Age, Race (White vs Non-white), Insurance Coverage (Medicare/Medicaid/Private/Self pay), “High volume hospital” status, Comorbidities	White male patients with private insurance and fewer comorbidities were most likely to receive SCS implants than other demographics and were most likely to be implanted at “High volume” hospitals
Wondwossen et al	US Military Health System (MHS) 2017 to January 2020	Inpatient and Outpatient	Active military or military retirees who identified as either Black or non-Latinx White, comorbidities, number of pain procedures received	Non-Latinx white patients were more likely to receive SCS overall and earlier in their treatment course than Black patients, although this difference became insignificant for individuals who had received 3 + pain procedures
Labaran et al	PearlDiver Patient Records Database 2007 to 2014	Inpatient and Outpatient	Paddle lead SCS implants, Age, Race, Region, Insurance Status (Medicare/Private-payer)	Factors including White race, female sex, Medicare coverage and Southern region in the age group 65-84y were more closely associated with SCS receipt with varying degrees of reported significance

## Discussion

The findings of our review suggest consistency in the following trends; SCS is more often completed in White patients vs other minorities in the outpatient setting, and more often in private-payer insurance holders and Medicare holders than Medicaid, dual Medicare/Medicaid holders, and self-pay patients. Given that Medicaid eligibility requires set household income capacities, it is reasonable to suggest an association between patient socioeconomic status, likelihood of carrying Medicaid/Medicare, and SCS implementation. In the inpatient setting, Black patients were found to receive SCS more frequently than White patients with all other variables removed, including insurance coverage. More SCS was performed for Black and Hispanic patients in the lowest income quartiles, however, income alone was not found to be significant when statistically isolated from insurance coverage in the inpatient setting.

Race was a significant independent variable of SCS receipt in all reviewed articles. SCS was completed more in White patients than any other minority observed with significant differences persisting even when all other differences were accounted for in the outpatient setting. The racial differences persisted even into veteran healthcare with White populations again receiving outpatient SCS at a higher rate than Black populations [12]. Wondwossen et al. did show that statistical significance between SCS discrepancy disappeared in those who had already received 3 or more spine procedures [12]. The majority of SCS are placed in the outpatient setting in modern pain management practices, however, inpatient SCS placement does occur. Orhurhu et al. documented that Black and Hispanic patients used SCS at a higher rate than their White counterparts while inpatient, in stark contrast to most other studies finding the opposite of this in the outpatient setting [11]. This discrepancy could be due to a variety of factors such as clinician racial biases, differences between treatment options offered for similar medical problems, delayed care in minority patients or differences in healthcare attitudes between racial groups [11]. It may be reasonable to assume that different attitudes toward pursuit of more or less aggressive medical care may lead to varying SCS utilization over time between observed groups. The inverted demographic trends between inpatient and outpatient SCS utilization are not easily explained. It has also been postulated that Black and minority candidates may be considered at lower rates for SCS due to a higher medical comorbidity burden, however, the inequity between SCS usage persists even when adjusted for medical comorbidities [8]. Furthermore, Jones et al. found that certain comorbidities such as diabetes, depression, nicotine dependence, and obesity were more likely to receive SCS than those without them [8]. Discrepancy between SCS utilization in the inpatient and outpatient setting between patient demographics is not fully understood but is multifactorial and likely involves some degree of healthcare associated bias.

Insurance plans and payer coverage are complex and controversial aspects of American healthcare. Most articles that reviewed insurance type concluded that private insurance conferred higher utilization of SCS receipt, often followed by Medicare and finally Medicaid. Labaran et al. did suggest that Medicare recipients may have received SCS at a higher rate in their observed population with a greater increase in incidence during the observation period [13]. Higher income is often subjectively associated with higher quality private healthcare insurance coverage. It may be reasonable to assume then that individuals in the highest income groups would be more likely to pursue SCS. Orhurhu et al. contest this notion by showing that in their observed population, household income was not independently associated with SCS implementation in the inpatient setting [11]. The authors did find that private insurance providers had a strongly positive association with SCS usage. Ovrom et al. also found that patients who underwent SCS in Black and Hispanic populations were more likely to be in the bottom quartile for median household income, unlike White patients who were evenly distributed among income quartiles [6]. Medicare was found to be the primary insurer across all racial groups. These studies may suggest that regardless of any linkage between higher SES and privatized insurance, those who had insurance plans that were less likely to cover SCS were less likely to pursue this therapy despite potentially more expendable income. In other terms, households whose insurance did not reimburse for SCS chose not to pursue SCS regardless of quartile income. These findings suggest that patients may often view insurance “coverage” as highly important and may be unwilling to self-pay for SCS, even if they are in the highest quartile of household earners and presumably able to afford the procedure with personal finances. Given that our study pools inclusion criteria across three commonly used patient databases and provides insight into a variety of patient cohorts, it is reasonable to postulate that insurance payout is a contributing factor for device placement. These pooled studies suggest consistent trends of a gap in standards of healthcare across different racial and socioeconomic subgroups may be due in some part to insurance differences, although the extent of which is difficult to accurately assess. For example, while studies such as Orhurhu et al. stratify patient cohorts into quartiles based on household income, more specific discussions can be had about the demographics of each quartile [11]. Further data collection and other variables pertaining to household members would help elucidate a better understanding of the decision-making process for patients who choose to accept or decline spinal cord stimulation as a treatment.

Our study has also pooled several reviews that consider geographic/regional implications in healthcare delivery, such as Missios et al. who stratified data in four different states, or Labaran et al. and Ovrom et al. who stratified data into regional sections of the USA: South/West/Midwest/Northeast [5, 6, 13]. Labaran et al. suggested a higher predominance of SCS therapy in the South region, which was acknowledged as per capita the largest population for private and Medicare databases [13]. Ovrom et al. reiterated this finding, confirming that the majority of inpatient SCS placement occurred in large Southern teaching hospitals in urban areas [6]. On average, hospitals in the Western region were associated with higher charges than those in the other US regions. The former study by Missios et al. suggests a predominance of SCS therapy in male patients in high-volume hospitals with fewer comorbidities, though does not address any data-based differences in therapy rates across the 4 states that were studied. In the larger context of such studies, further research might lean into state-by-state analysis of differences in pain management practices and the rate of SCS across these regions. Possible confounding variables such as climate, terrain, infrastructure and ease of transportation can all play roles in averages of a demographics’ physical activity, pain experience and lifestyle. Furthermore, psychosocial and cultural attitudes towards surgical treatment and Western medicine would provide useful information in the context of SCS rates in these populations. While many studies successfully analyze the completion rate of these procedures, understanding the needs and perspectives of minority patients or patients in lower socioeconomic quartiles will help future caregivers and policy makers consider how to modify their practices to address these gaps. Of note, it is important to stratify in future studies the reasons why SCS is or is not utilized in any given patient. For example, whether SCS was 1.) Not offered as a therapy by the provider vs 2.) Patient rejection of the proposed procedure vs 3.) Patient interest + physician-offered but not covered by insurance. Understanding other financially driven reasons for patient rejection such as the ability to take time off work/household support/family needs is crucial in finding ways to remediate the disparities observed. Such information may be difficult to ascertain, but such decisions are undoubtedly contributing to many patient’s decisions to pursue SCS and may differ significantly between socioeconomic and racial groups.

Another key consideration that the prior studies have not explored is the per capita prevalence of physicians who are appropriately certified in pain management and or providers credentialed to place spinal cord stimulators. Rural landscapes with known limitations in healthcare availability and/or specialists may, unsurprisingly, have drastically lower rates of satisfactory pain management and SCS procedure completion rates. Further research is needed in these domains to acquire a more complete understanding of why these disparities exist so that they can be better accounted for and addressed in the future of pain management. As a closing consideration, it is also worth noting that the timeline for the examined data often spans very discrete time intervals, typically only several years. For example, Orhurhu et al. focuses on a cohort explicitly between 2011–2015, while Wondwossen et al. examines data points exclusively between 2017–2020 [11, 12]. In the context of segmented data such as this, the importance of continued meta-analyses becomes crucial in observing patient statistics not only laterally, through as many relevant cohorts as possible, but longitudinally across generations to better understand the long term medical and societal trends.

## Conclusion

There is compelling evidence to support that White and private or Medicare insured populations are more frequent recipients of SCS and may undergo SCS therapy earlier in their spine care than minority and more poorly insured groups. In addition, there is also evidence to suggest that White patients receive SCS at a higher rate than all other races, even when race is stratified from other variables. We suspect that there are many factors widening the disparity of care between the assessed groups, including inherent healthcare bias. Our review, however, did not assess outcome inequalities between race and SCS receipt and therefore we do not suggest wide prescription to grossly expand SCS usage in minority and low SES populations preferentially. Clinicians should not attempt to remedy the current known disparities by offering SCS at higher rates to minorities and those of low SES who were otherwise not considered SCS candidates. Rather, patients should be assessed individually for medical appropriateness for SCS and have a shared decision-making process regarding their desire for this form of treatment. Each clinician should undergo introspection and training to understand their own socioeconomic and racial biases, particularly in regard to treatment options such as SCS. There are many complex personal and socioeconomic factors involved in the decision to pursue SCS and these relationships are often not well discussed or reported. As documented in this report, there is a body of literature that describes a known disparity between race, SES and SCS therapy. The cause of this disparity is likely multifaceted and poorly understood, however, likely involves significant healthcare racial bias. Further work and research need to be completed to fully understand this disparity and eliminate the associated racial bias to deliver inclusive and personalized healthcare.

## Key References

- Ovrom E, Hagedorn JM, Bhandarkar A, Bydon M. Racial disparities in the cost of inpatient spinal cord stimulator surgery among patients in the 2016–2018 National Inpatient Sample. *Journal of Clinical Neuroscience*. 2022;98:189–193. 10.1016/j.jocn.2022.02.019
  - Ovrum et al. provide key insights into the SCS utilization within the NIS database which expands on previous work such as Orhurhu et al. and others. Race, regional differences and income stratification were further characterized under this report which allows for more complete discussions regarding SCS among varying demographics.
- Jones MR, Orhurhu V, O’Gara B, et al. Racial and socioeconomic disparities in spinal cord stimulation among the Medicare population. *Neuromodulation: Technology at the Neural Interface*. 2021;24(3):434–440. 10.1111/ner.13373
  - Jones et al. provide the most recent analysis of the Medicare Limited Data sets with extraction of SCS related data from the outpatient and ambulatory settings. This analysis permits contrast from strictly inpatient databases and promotes further discussion regarding potential influences that may differ between the two settings.
- Wondwossen Y, Patzkowski MS, Amoako MY, et al. Spinal cord stimulator inequities within the US Military Health System. *Neuromodulation: Technology at the Neural Interface*. Published online April 2023. 10.1016/j.neurom.2023.03.008
  - Wondwossen et al. provides exclusive analysis of SCS in the active and retired US military population which possibly reduces some variables such as insurance coverage and provides a more homogenous population. Evaluation of SCS inequity in this population is a valuable comparator to civilian populations and provides some insight into inequities that persist despite the two groups.

## Data Availability

No datasets were generated or analysed during the current study.

## References

[1] Fiscella K, Franks P, Gold MR, Clancy CM. Inequality in quality. JAMA. 2000;283(19):2579. 10.1001/jama.283.19.2579. 10815125 10.1001/jama.283.19.2579

[2] Stepanikova I, Oates GR. Perceived Discrimination and Privilege in Health Care: The Role of Socioeconomic Status and Race. Am J Prev Med. 2017;52(1S1):S86–94. 10.1016/j.amepre.2016.09.024. 27989297 10.1016/j.amepre.2016.09.024 PMC5172593

[3] Janevic MR, McLaughlin SJ, Heapy AA, Thacker C, Piette JD. Racial and socioeconomic disparities in disabling chronic pain: Findings from the Health and Retirement Study. J Pain. 2017;18(12):1459–67. 10.1016/j.jpain.2017.07.005. 28760648 10.1016/j.jpain.2017.07.005 PMC5682226

[4] Dahlhamer J, Lucas J, Zelaya C, et al. Prevalence of chronic pain and high-impact chronic pain among adults — United States, 2016. MMWR Morb Mortal Wkly Rep. 2018;67(36):1001–6. 10.15585/mmwr.mm6736a2. 30212442 10.15585/mmwr.mm6736a2 PMC6146950

[5] Missios S, Rahmani R, Bekelis K. Spinal cord stimulators: Socioeconomic disparities in four US states. Neuromodulation: Technol Neural Interface. 2014;17(5):451–6. 10.1111/ner.12101. 10.1111/ner.12101 23924155

[6] Ovrom E, Hagedorn JM, Bhandarkar A, Bydon M. Racial disparities in the cost of inpatient spinal cord stimulator surgery among patients in the 2016–2018 National Inpatient Sample. J Clin Neurosci. 2022;98:189–93. 10.1016/j.jocn.2022.02.019. 35189543 10.1016/j.jocn.2022.02.019

[7] Vallejo R, Bradley K, Kapural L. Spinal cord stimulation in chronic pain: mode of action. Spine (Phila Pa 1976). 2017;42(Suppl 14):S53–60. 10.1097/BRS.0000000000002179. 28368982 10.1097/BRS.0000000000002179

[8] Jones MR, Orhurhu V, O’Gara B, et al. Racial and socioeconomic disparities in spinal cord stimulation among the Medicare population. Neuromodulation: Technol Neural Interface. 2021;24(3):434–40. 10.1111/ner.13373. 10.1111/ner.13373 33723896

[9] Caylor J, Reddy R, Yin S, Cui C, Huang M, Huang C, Ramesh R, Baker DG, Simmons A, Souza D, Narouze S, Vallejo R, Lerman I. Spinal cord stimulation in chronic pain: evidence and theory for mechanisms of action. Bioelectron Med. 2019;5:12. 10.1186/s42234-019-0023-1. 31435499 10.1186/s42234-019-0023-1 PMC6703564

[10] Garcia K, Wray JK, Kumar S. Spinal cord stimulation. StatPearls [Internet]. April 24, 2023. https://www.ncbi.nlm.nih.gov/books/NBK553154/. Accessed 24 March 2024

[11] Orhurhu V, Gao C, Agudile E, et al. Socioeconomic disparities in the utilization of spinal cord stimulation therapy in patients with chronic pain. Pain Pract. 2020;21(1):75–82. 10.1111/papr.12936. 32654360 10.1111/papr.12936

[12] Wondwossen Y, Patzkowski MS, Amoako MY, Lawson BK, Velosky AG, Soto AT, Highland KB. Spinal Cord Stimulator Inequities Within the US Military Health System. Neuromodulation. 2024;27(5):916–22. 10.1016/j.neurom.2023.03.008. 38971583 10.1016/j.neurom.2023.03.008

[13] Labaran L, Bell J, Puvanesarajah V, et al. Demographic trends in paddle lead spinal cord stimulator placement: Private Insurance and Medicare beneficiaries. Neurospine. 2020;17(2):384–9. 10.14245/ns.1938276.138. 32054146 10.14245/ns.1938276.138 PMC7338957

